# Exploring the mechanism of Danshen in treating Kawasaki disease through network pharmacology and molecular docking approaches

**DOI:** 10.1097/MD.0000000000044163

**Published:** 2025-08-29

**Authors:** Aiyuan Cai, Huishi Ye, Yuanhong Lin, Meiping Shi, Zhiwei Li, Jinyun Li, Guangliang Luo, Yanfang Huang, Ciai Lai

**Affiliations:** a Shenzhen Hospital (Futian) of Guangzhou University of Chinese Medicine, Shenzhen, Guangdong Province, China; b Dongguan Hospital of Guangzhou University of Chinese Medicine, Dongguan, Guangdong Province, China; c Second Clinical Medical College, Guangzhou University of Chinese Medicine, Guangzhou, Guangdong Province, China; d Xi’an Jiaotong University, Xi’an, Shaanxi Province, China; e Acupuncture Rehabilitation Clinical College, Guangzhou University of Chinese Medicine, Guangzhou, Guangdong Province, China.

**Keywords:** anti-inflammatory, antiplatelet, Danshen, Kawasaki disease, network pharmacology

## Abstract

This study aims to identify the primary active components of Danshen (Salvia miltiorrhiza) and explore the potential mechanisms underlying its therapeutic effect on Kawasaki disease (KD). Active components of Danshen and their action targets were screened using traditional Chinese medicine systems pharmacology and SwissTargetPrediction databases. KD-related targets were retrieved from Online Mendelian Inheritance in Man, Pharmacogenomics Knowledge Base, and GeneCards databases. Intersection targets between KD-related genes and the main active components of Danshen were identified. The protein–protein interaction network for intersecting targets of Danshen and KD was constructed using Search Tool for the Retrieval of Interacting Genes/Proteins database and Cytoscape software, and core genes were identified. Gene ontology functions and Kyoto Encyclopedia of Genes and Genomes pathway enrichment analysis of Danshen–KD intersecting targets were performed using database for annotation, visualization, and integrated discovery database. Molecular docking was conducted using Autodock software. A total of 65 active components of Danshen and 219 drug-related target proteins were identified, establishing 1308 KD-related human genes. The intersection yielded 70 potential drug targets associated with KD. Construction of the protein–protein interaction network for these common targets revealed 7 core genes: IL6, TNF, AKT1, BCL2, STAT3, CASP3, and TP53. Kyoto Encyclopedia of Genes and Genomes enrichment analysis indicated that these targets were primarily enriched in pathways such as Pathways in Cancer, Lipid and Atherosclerosis, and Fluid Shear Stress and Atherosclerosis. Molecular docking results demonstrated that core active components of Danshen, including luteolin, tanshinone IIA, NSC 122421, (Z)-3-[2-[(E)-2-(3,4-dihydroxyphenyl)vinyl]-3,4-dihydroxy-phenyl]acrylic acid, and cryptotanshinone, could regulate the 7 core genes within these pathways. Danshen treats Kawasaki disease through a multicomponent, multi-target approach, potentially involving anti-inflammatory, antiapoptotic, antitumor, and anti-atherosclerotic mechanisms. This study provides scientific evidence for the use of Danshen in treating Kawasaki disease and offers new clues for targeting in the treatment of KD with traditional Chinese medicine.

## 1. Introduction

Kawasaki disease (KD) is an acute, systemic immunovasculitis of unknown etiology, predominantly affecting young children, and is the primary cause of acquired heart disease in children in developed countries.^[1]^ The exact etiology of the disease remains uncertain, with significant geographical and ethnic disparities. Over the past few decades, there has been a global increase in the incidence of KD, with higher rates observed in Asian populations compared to Caucasian children.^[2]^ KD is mostly diagnosed by looking at the symptoms and ruling out other illnesses. Common symptoms include fevers that come and go, swollen lymph nodes in the neck, stuffy eyes, and a rash that looks different on each person. KD involves systemic medium and small vessels, with pathogenic factors activating a systemic immune response, leading to a cascade of cytokines that ultimately cause endothelial damage and dysfunction. Coronary artery lesions are the most common complication, occurring in about 25% of untreated children and potentially leading to thrombosis, myocardial ischemia, infarction, or even death.^[3,4]^

The pharmacological treatment of KD primarily focuses on anti-inflammatory actions and preventing aneurysms and thrombosis caused by platelet aggregation. High-dose immunoglobulin combined with aspirin is the standard initial treatment, significantly reducing the incidence of coronary artery aneurysms. Intravenous immunoglobulin is a passive immunotherapeutic agent that boosts immune function, stops vascular immune inflammation, lowers levels of inflammatory factors, makes immune regulatory cells give off negative feedback, and helps immune disorders. However, 10% to 20% of patients are insensitive to initial immunoglobulin therapy.^[3]^ Aspirin, a nonsteroidal anti-inflammatory drug, has potent anticoagulant and anti-inflammatory effects. Still, there is no evidence that acetylsalicylic acid reduces the risk of coronary artery lesions, and the optimal dosage remains controversial.^[5]^ Long-term or excessive use can lead to serious adverse drug reactions, such as gastrointestinal bleeding and hepatic-renal damage, impacting the long-term quality of life of children. Due to the dependence on clinical symptoms for diagnosis, the timing for initiating treatment in atypical cases remains unclear, and there are instances of drug insensitivity and severe adverse reactions, highlighting the need for more effective and safe adjunctive treatments.

Traditional Chinese medicine (TCM) has a complex system of multicomponent, multi-target actions and often demonstrates synergistic effects in clinical use. KD, based on its common clinical manifestations, falls under the category of “wen disease” in TCM. Clinical treatments often follow the principle of Wei–Qi–Ying–Xue, focusing on clearing heat, activating blood, and nourishing yin. Danshen is a traditional Chinese herb that is known for getting blood moving and preventing blood clots. It has also been shown to have strong anti-inflammatory, antioxidant, atherosclerotic, and tumor-fighting properties.^[6]^ Hui Chen et al^[7]^ found that tanshinone, an active component of Danshen, has superior anti-inflammatory and antiplatelet effects compared to aspirin and immunoglobulins. However, the specific mechanisms underlying these effects are not yet clear. Network pharmacology offers an approach to studying the interactions between drugs, components, targets, and diseases. This study employs network pharmacology to explore the potential mechanisms of Danshen in treating KD, providing a reference for adjunctive clinical therapy.

## 2. Methods

### 2.1. Selection of main active components of Danshen and their target proteins

The traditional Chinese medicine systems pharmacology database and analysis platform (TCMSP, https://www.tcmsp-e.com/tcmsp.php) was used to collect chemical components of Danshen. Compounds were analyzed based on pharmacokinetic distribution, absorption, metabolism, and excretion parameters. Two thresholds were set: Oral bioavailability ≥ 30% and drug-likeness ≥ 0.18 to select the main active components of Danshen and identify potential target proteins. For compounds without target information in the TCMSP database, the CanonicalSMILES sequences obtained from the PubChem database were used for protein target prediction in the SwissTargetPrediction database (http://www.swisstargetprediction.ch). These identified target proteins were further searched in the UniProt database (https://www.uniprot.org/) to find corresponding human target genes.

### 2.2. Selection of KD-related targets

KD-related targets were retrieved using the Online Mendelian Inheritance in Man (http://www.omim.org), Pharmacogenomics Knowledge Base (https://www.pharmgkb.org), GeneCards (http://www.genecards.org), and National Center for Biotechnology Information (https://www.ncbi.nlm.nih.gov) databases with the keyword “Kawasaki disease.” In the GeneCards database, targets with a relevance score > 10 were selected. The targets obtained were then screened, integrated, and redundant targets were removed to establish a KD-related target set. This study was conducted in accordance with the revised Declaration of Helsinki (2013).

### 2.3. Venn analysis of potential targets of Danshen for KD

A Venn diagram analysis of KD-related targets and target sets of the main active components of Danshen was conducted using Bioinformatics (www.bioinformatics.com.cn). This identified intersecting targets for Danshen and KD, leading to the construction of a “Danshen–active component–target–KD” network diagram.

### 2.4. Construction of protein–protein interaction (PPI) network for Danshen–KD intersecting targets and selection of key targets

The intersecting targets for Danshen–KD were uploaded to the STRING 11.5 online database (http://string-db.org), with “Homo sapiens” as the species and interaction threshold set to “medium confidence (0.400).” This provided information on PPIs, which were imported into Cytoscape 3.9.1 software to construct a PPI network diagram. Topological analysis of the PPI network was conducted using the CytoNAC plugin in Cytoscape 3.9.1, which included betweenness, closeness, degree, and eigenvector. The top 10 nodes in each topological analysis method were identified, and the intersecting genes were deemed core genes.

### 2.5. Gene ontology (GO) function and Kyoto Encyclopedia of Genes and Genomes (KEGG) pathway analysis of Danshen–KD intersecting targets

The intersecting targets were uploaded to the Metascape database (http://metascape.org) with “Homo sapiens” as the species. GO function and KEGG pathway enrichment analyses were performed. *P*-values of the enrichment results were calculated and sorted in ascending order. Visualization of the results was conducted using the Bioinformatics online drawing platform (http://www.bioinformatics.com.cn).

### 2.6. Molecular docking verification of main active components of Danshen in treating KD and potential target proteins

Active components and potential target proteins with high degree values from the “Danshen–active component–target gene–KD” network and core genes from the PPI network were selected for molecular docking. Molecular structures of Danshen chemical components and target protein molecules were obtained from the PubChem and RCSB PDB databases, respectively, and converted into pdbqt format using AutoDock Tools 1.5.6 software for molecular docking. Visualization of molecular docking with higher docking scores was conducted using Pymol 2.6.0 software.

## 3. Results

### 3.1. Screening of active components of Danshen and prediction of their potential targets

**Figure 1.**
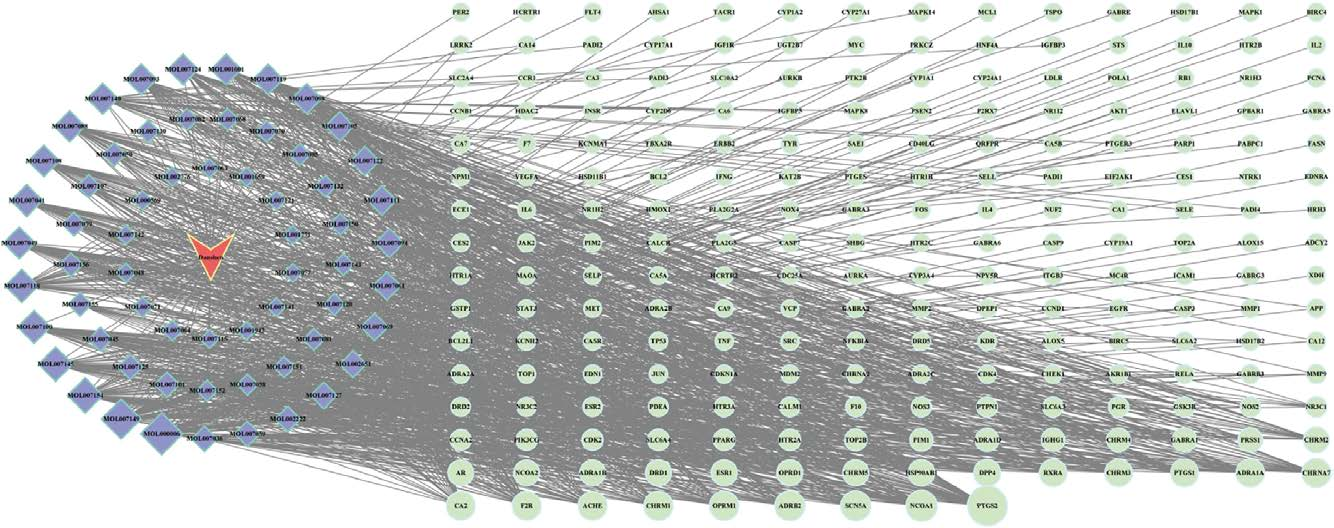
Predicted targets of Danshen potential action.

A total of 65 active components of Danshen were identified through the TCMSP database. Among these, 62 active components had potential targets identified via the TCMSP platform and SwissTargetPrediction database. After removing duplicate targets, 1041 potential target proteins were identified. These were standardized in the UniProt database, resulting in 219 distinct drug target proteins of Danshen (Fig. 1).

### 3.2. Determination of intersection targets of Danshen and Kawasaki disease

**Figure 2.**
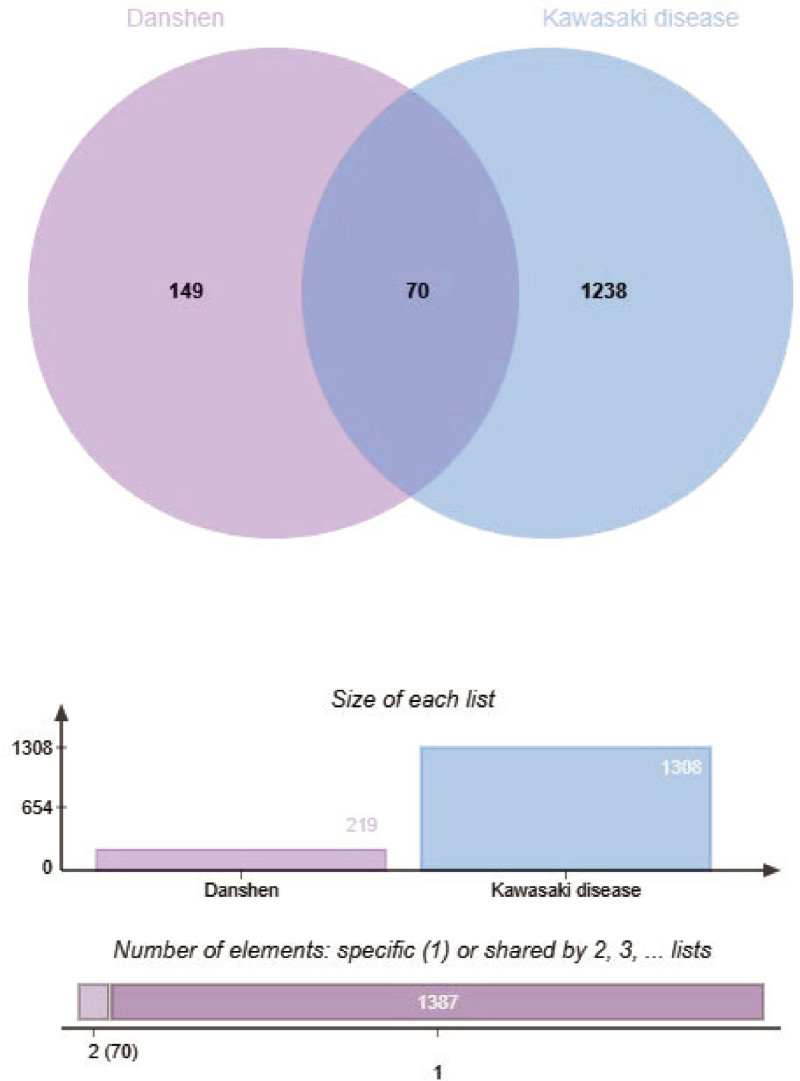
Intersection of genes related to major components of Danshen and Kawasaki disease.

1308 human genes related to Kawasaki disease were obtained from the Online Mendelian Inheritance in Man, Pharmacogenomics Knowledge Base, GeneCards, and National Center for Biotechnology Information databases. The intersection of 219 potential targets of Danshen and these 1308 KD-related genes yielded 70 genes (Table 1), indicating that 62 active components of Danshen are associated with these 70 potential KD targets (Fig. 2).

**Table 1:** List of common targets.

No.	Targets	No.	Targets	No.	Targets	No.	Targets	No.	Targets
1	AKT1	15	EDN1	29	IGF1R	43	MMP1	57	PTGS2
2	APP	16	EGFR	30	IGFBP3	44	MMP2	58	RELA
3	AR	17	ERBB2	31	IL10	45	MMP9	59	SCN5A
4	BCL2	18	ESR1	32	IL2	46	MYC	60	SELE
5	CASP3	19	ESR2	33	IL4	47	NCOA1	61	SELL
6	CASP9	20	F10	34	IL6	48	NFKBIA	62	SELP
7	CCND1	21	F7	35	INSR	49	NOS2	63	SRC
8	CCR1	22	FLT4	36	JUN	50	NOS3	64	STAT3
9	CD40LG	23	FOS	37	KDR	51	NR1H3	65	TBXA2R
10	CDKN1A	24	GSTP1	38	MAPK1	52	NR3C2	66	TNF
11	CYP3A4	25	HMOX1	39	MAPK14	53	P2RX7	67	TP53
12	DPP4	26	HNF4A	40	MAPK8	54	PIK3CG	68	VCP
13	DRD2	27	ICAM1	41	MDM2	55	PPARG	69	VEGFA
14	ECE1	28	IFNG	42	MET	56	PSEN2	70	XDH

### 3.3. Construction of PPI network and selection of key targets for Danshen–KD intersection targets

**Figure 3.**
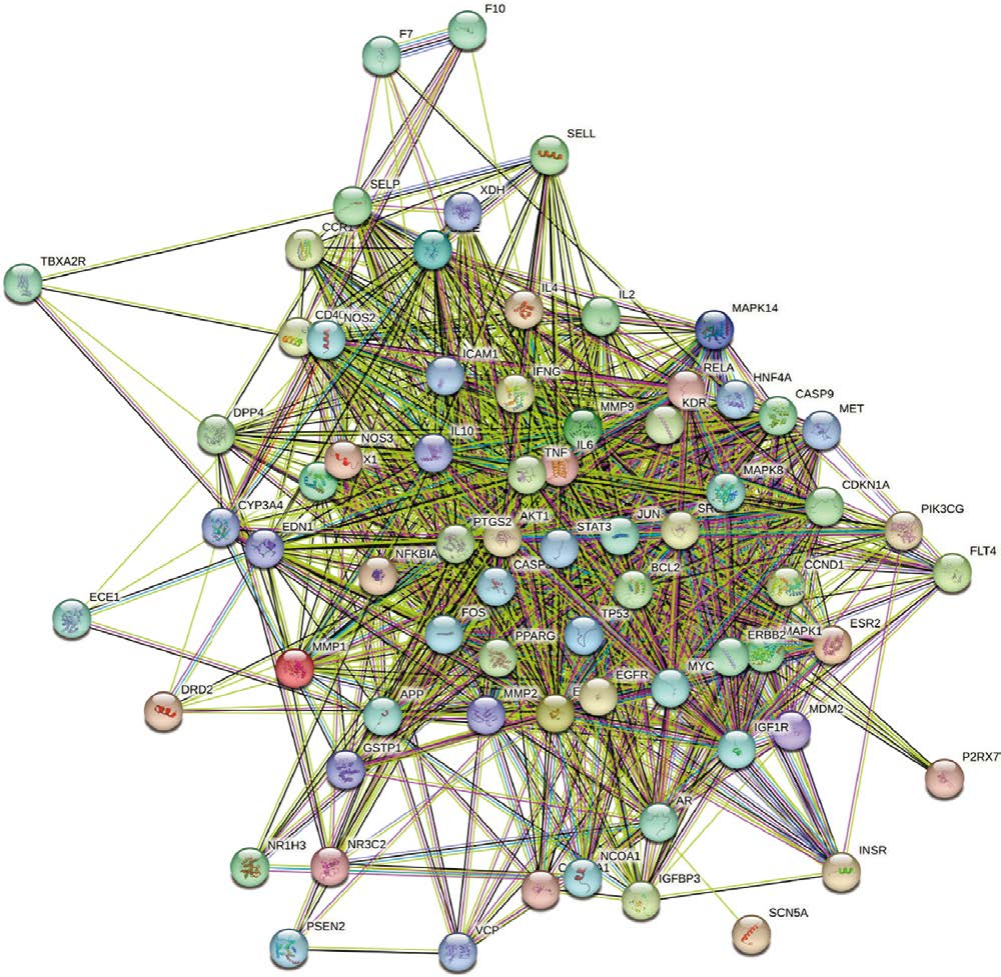
STRING-based protein–protein interaction (PPI) network. STRING = Search Tool for the Retrieval of Interacting Genes/Proteins.

**Figure 4.**
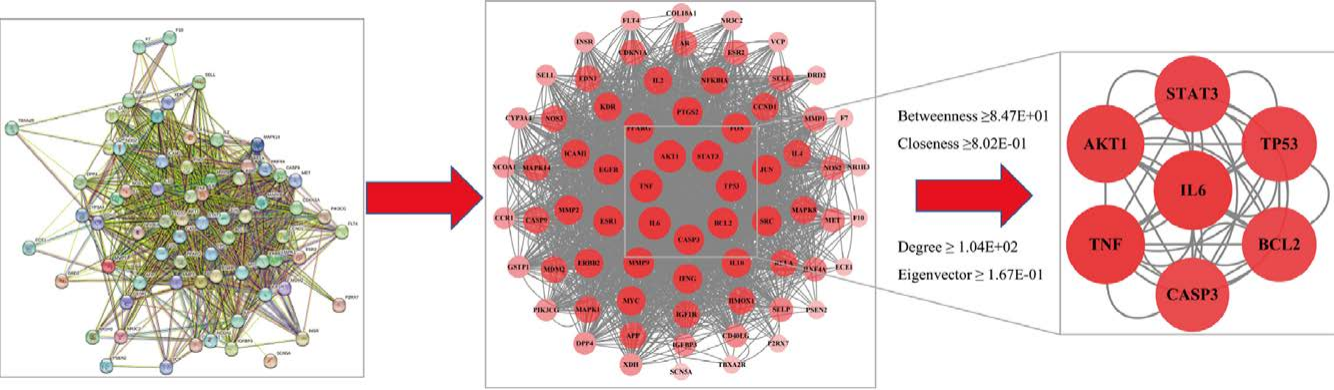
PPI network analysis diagram. PPI = protein–protein interaction.

A PPI network of 70 common target genes of Danshen and KD was constructed using the Search Tool for the Retrieval of Interacting Genes/Proteins database (Fig. 3). The network comprised 70 nodes and 1131 edges. The interaction strength of targets was indicated by degree values. Seven overlapping core genes were identified using betweenness, closeness, degree, and eigenvector topological analysis methods in Cytoscape’s CytoNAC plugin (Table 2). These core genes were IL6, TNF, AKT1, BCL2, STAT3, CASP3, and TP53 (Fig. 4), suggesting a significant association of these targets with Danshen treatment of KD.

**Table 2:** Topological property parameters of key targets in the treatment of Kawasaki disease by Danshen.

Targets	Key action genes	Degrees	CytoNAC-betweenness score	CytoNAC-closeness score	CytoNAC-eigenvector score
Interleukin-6	IL6	120	262.1914042	0.884615385	0.174421623
Tumor necrosis factor	TNF	120	201.7153498	0.884615385	0.175939411
RAC-alpha serine/threonine-protein kinase	AKT1	116	139.7129327	0.8625	0.174596339
Apoptosis regulator BCL2	BCL2	106	105.623307	0.811764706	0.168173954
Signal transducer and activator of transcription 3	STAT3	110	98.69847872	0.831325301	0.170927405
Caspase-3	CASP3	104	95.04303314	0.802325581	0.166824713
Cellular tumor antigen p53	TP53	108	84.69562062	0.821428571	0.168844461

### 3.4. Construction of the Danshen–active component–target–KD network

**Figure 5.**
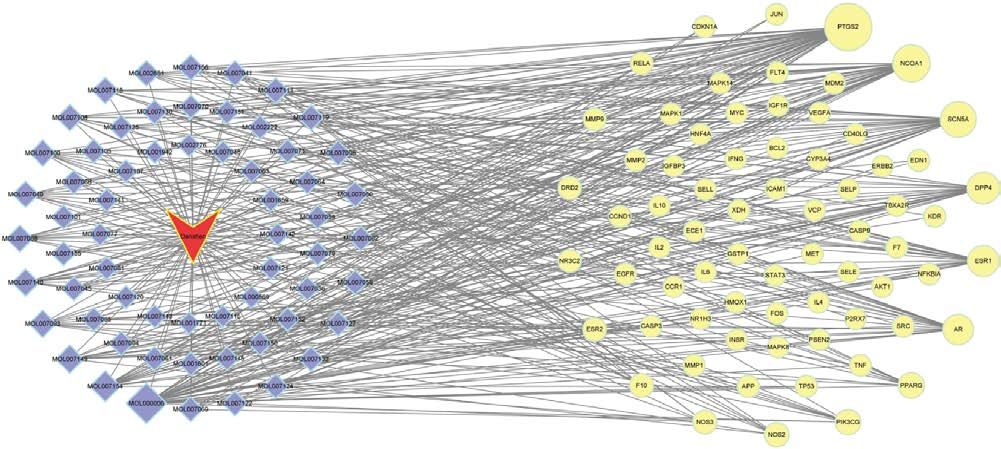
Network diagram of Danshen–component–target–Kawasaki disease.

The active components of Danshen and their KD-related targets were imported into Cytoscape to construct a “Danshen–active component–target–KD” network diagram (Fig. 5). The NetworkAnalyzer tool in Cytoscape was used to analyze network topological parameters, primarily focusing on degree values to determine important components and targets. The network contained 133 nodes and 362 edges. Luteolin, tanshinone IIA, NSC 122421, (Z)-3-[2-[(E)-2-(3,4-dihydroxyphenyl)vinyl]-3,4-dihydroxy-phenyl]acrylic acid, and cryptotanshinone were identified as the top 5 Danshen components in degree value, considered as potential core compounds for treating KD.

### 3.5. GO function and KEGG pathway enrichment analysis of key targets for Danshen–KD

**Figure 6.**
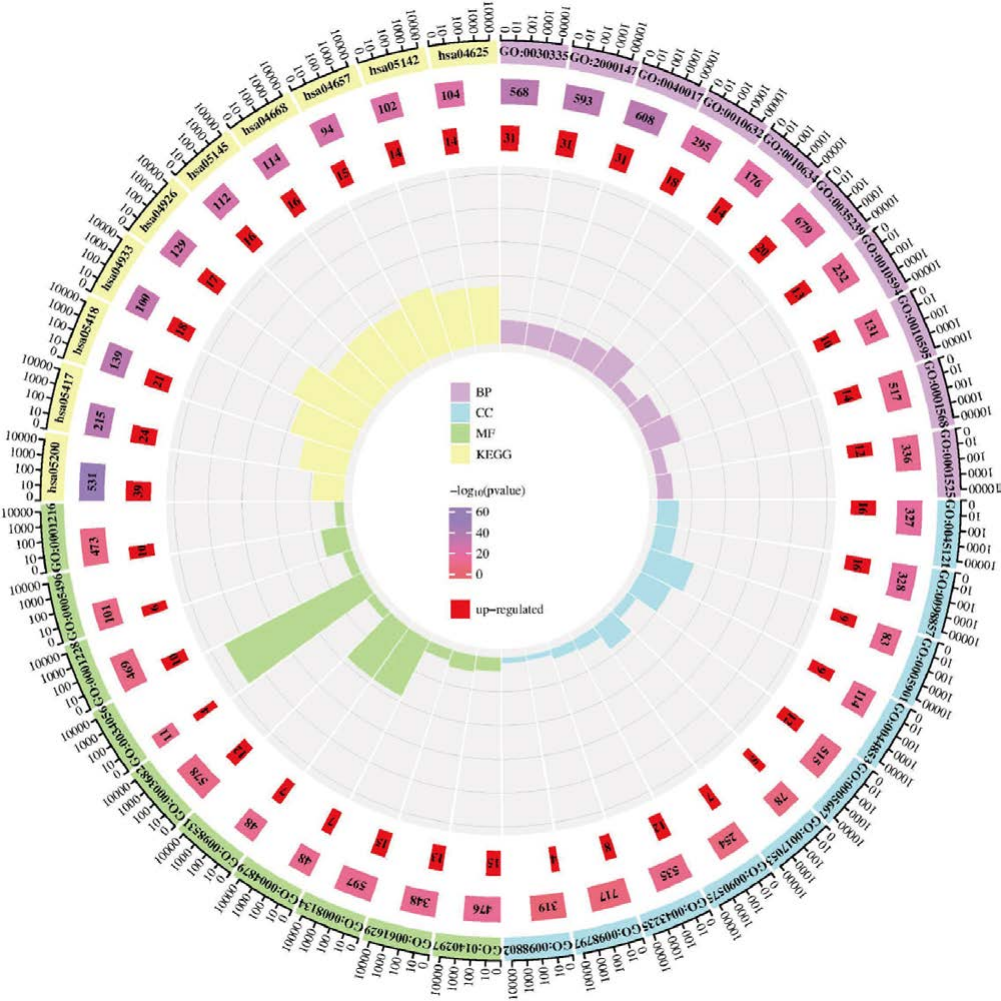
GO and KEGG enrichment circle diagram. GO = gene ontology, KEGG = Kyoto Encyclopedia of Genes and Genomes.

According to FDR results, the intersection targets of Danshen–KD were primarily involved in biological processes such as positive regulation of cell migration, cell motility, and locomotion, suggesting Danshen influence on KD through these processes. Cellular components included membrane raft, membrane microdomain, caveola, etc. Molecular functions mainly involved DNA-binding transcription factor binding, RNA polymerase II-specific DNA-binding transcription factor binding, transcription factor binding, etc. KEGG pathway enrichment analysis revealed that Danshen–KD intersecting targets were mainly concentrated in pathways in cancer, lipid and atherosclerosis, fluid shear stress and atherosclerosis, etc (Fig. 6).

### 3.6. Molecular docking predictions of potential core compounds of Danshen and key targets

**Figure 7.**
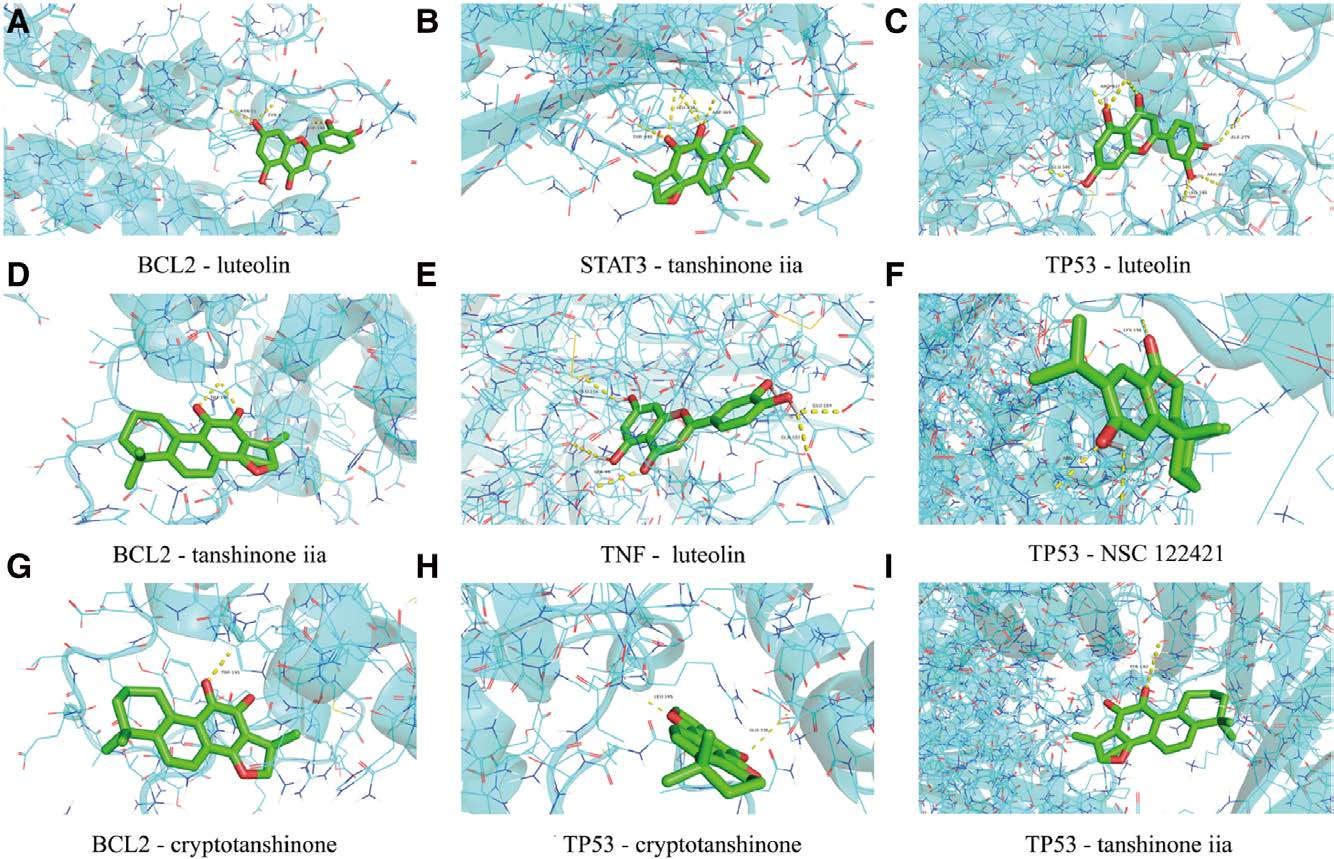
Molecular docking model of major active components of Danshen with potential targets.

The top 5 core active components identified in the “Danshen–active component–target gene–KD” network—luteolin, tanshinone IIA, NSC 122421, (Z)-3-[2-[(E)-2-(3,4-dihydroxyphenyl)vinyl]-3,4-dihydroxy-phenyl]acrylic acid, cryptotanshinone—were docked with the 7 core genes IL6, TNF, AKT1, BCL2, STAT3, CASP3, and TP53 using AutoDock Tools software. Binding affinities below −4.25 kcal/mol indicate some binding activity, while those below −5.0 kcal/mol suggest good binding activity, and values below −7.0 kcal/mol indicate strong binding. The molecular docking results showed that all 7 core targets had binding affinities below −5.0 kcal/mol with the 5 core active components, indicating good binding activity. The lower the binding energy, the stronger the binding affinity. Core targets and components with binding energies lower than −8.0 kcal/mol were selected for molecular docking visualization analysis using Pymol software, including BCL2 with luteolin, tanshinone IIA, cryptotanshinone; STAT3 with tanshinone IIA; TNF with luteolin; TP53 with cryptotanshinone, luteolin, NSC 122421, and tanshinone IIA (Fig. 7).

## 4. Discussion

Using both Western medicine and TCM to treat Kawasaki disease has been shown to improve symptoms more often than using only Western medicine.^[8]^ This can shorten the course of the disease and lower drug side effects.^[8]^ However, the current clinical treatment of KD is primarily based on Western medicine, with TCM serving only as an adjunct. The potential mechanisms of TCM for treating KD require further research. Western medical treatment focuses on anti-inflammatory and antiplatelet therapies, and the anti-inflammatory and antiplatelet activities of Danshen have been validated in experiments.^[7]^

This study, utilizing network pharmacology and molecular docking, explores the mechanism of Danshen in treating KD, promoting the clinical use of TCM. Our results show that luteolin, tanshinone IIA, NSC 122421, (Z)-3-[2-[(E)-2-(3,4-dihydroxyphenyl)vinyl]-3,4-dihydroxy-phenyl]acrylic acid, and cryptotanshinone are the main active components of Danshen in treating KD. The immunopharmacological activity of luteolin has been confirmed in many studies, showing anti-inflammatory, antioxidative, apoptosis-regulating, autophagy-increasing, and lipid metabolism-involved effects in cardiovascular diseases,^[9]^ in addition to its potent antitumor activity. Tanshinone IIA, a lipophilic component of Danshen, effectively exerts anti-inflammatory effects by blocking the upregulation of pro-inflammatory mediators such as tumor necrosis factor-alpha and interleukins.^[10]^ Maione F et al^[11]^ demonstrated that tanshinone IIA inhibits rat platelet aggregation and activation through the ERK-2 signaling pathway, suggesting its efficacy in improving blood viscosity and microcirculation, and in preventing cardiovascular diseases. Cryptotanshinone also exhibits a variety of pharmacological activities, including anticancer, anti-inflammatory, immunomodulatory, neuroprotective, and anti-fibrotic effects,^[12]^ with mechanisms involving multiple signaling pathways such as PI3K-Akt, p53, Jak-STAT, NF-kappa B, MAPK, FoxO, and TNF. IL6, TNF, AKT1, BCL2, STAT3, CASP3, and TP53 are key targets of action. IL6 plays an important role in immune regulation, cellular metabolism, and inflammatory response, with its inflammatory signaling activation causing endothelial cell damage.

In the acute phase of KD, IL6 levels are significantly elevated and return to normal after standard immunoregulatory treatment.^[13]^ Elevated IL6 levels are also associated with the incidence of coronary artery lesions and resistance to immunoglobulin therapy.^[14]^ The PI3K/AKT signaling pathway is crucial in regulating cell proliferation, differentiation, metabolism, and apoptosis, with TNF as an upstream site. Activation of this pathway directly phosphorylates downstream apoptotic target proteins or encodes the expression of apoptosis-related proteins, including BCL2 and CASP3, mediating cellular biological processes. Gao et al^[15]^ showed that Danshen activates AKT1 protein expression, significantly reducing necrosis and apoptosis in rat brain tissues. Tucka J. et al^[16]^ demonstrated that AKT1 activation inhibits apoptosis of vascular smooth muscle cells during atherosclerotic plaque formation. TP53, a tumor suppressor gene, plays a key role in regulating multiple cellular processes, including the cell cycle, oxidative stress, DNA replication and repair, apoptosis, and autophagy.^[17]^

STAT3 is a protein composed of 770 amino acids that is highly active in both cancer and non-cancer cells, playing a significant role in inhibiting the expression of key immune activation regulators and promoting the production of immunosuppressive factors.^[18]^ Furthermore, KEGG pathway enrichment analysis indicates that the intersecting targets of Danshen–KD are primarily concentrated in pathways in cancer, lipid and atherosclerosis, fluid shear stress and atherosclerosis, etc. Lequain Hippolyte reported a certain association between 2 adult KD patients and malignant tumors.^[19]^ As arterial lesions are the most common complication of KD,^[3]^ the lipid and atherosclerosis, fluid shear stress, and atherosclerosis pathways are closely related to arterial lesions,^[20,21]^ suggesting that these pathways might be involved in KD pathogenesis by affecting arterial lesions.

## 5. Conclusions

This study employed network pharmacology to investigate the mechanism by which Danshen treats Kawasaki disease. The findings suggest that tanshinone, a crucial constituent of Danshen, may alleviate the symptoms of Kawasaki disease by suppressing inflammatory responses, combating cancer, and mitigating atherosclerosis through various targets and pathways. The primary targets for intervention identified in this study were IL6, TNF, AKT1, BCL2, STAT3, CASP3, and TP53.

## Author contributions

**Conceptualization:** Aiyuan Cai.

**Data curation:** Aiyuan Cai.

**Formal analysis:** Meiping Shi, Zhiwei Li, Guangliang Luo, Yanfang Huang.

**Investigation:** Huishi Ye, Zhiwei Li, Jinyun Li.

**Methodology:** Huishi Ye, Yuanhong Lin, Yanfang Huang.

**Project administration:** Yuanhong Lin.

**Resources:** Meiping Shi, Jinyun Li, Guangliang Luo.

**Supervision:** Ciai Lai.

**Writing – original draft:** Aiyuan Cai.

**Writing – review & editing:** Ciai Lai.
